# Magnesium application partially reversed the negative effects of mulching on rhizosphere nitrogen cycling in a *Phyllostachys praecox* forest

**DOI:** 10.3389/fpls.2025.1670128

**Published:** 2025-10-08

**Authors:** Hong Zhao, Xiaobao Kuang, Yi Li, Tian Li, Tian Wang, Lin Yu, Congcong Xia, Huanhuan Dong, Jiacheng Shen, Bojie Fu, Hanchang Zhou

**Affiliations:** ^1^ Jiangxi Communications Investment Maintenance Technology Group Co., Ltd., Nanchang, China; ^2^ The Bamboo Institute, Jiangxi Academy of Forestry, Nanchang, China; ^3^ Jiangxi Jinggangshan Bamboo Forest Ecosystem National Observation and Research Station, Jinggangshan, Jiangxi, China; ^4^ The Gardens Institute, Jiangxi Academy of Forestry, Nanchang, China; ^5^ The Research Centre for Eco-environmental Sciences, Chinese Academy of Sciences, Beijing, China

**Keywords:** Lei-bamboo, mulching, rhizosphere nitrogen cycling, nxrA, magnesium

## Abstract

Mulching is the practice of covering soil with a layer of organic or inorganic material. While this process is often used in bamboo forests to increase yield, it has also been found to lead to bamboo degradation, especially in *Phyllostachys praecox* (Lei-bamboo) forests. Studies suggest that mulching might accelerate degradation by altering rhizosphere nitrogen cycling, a process likely influenced by the depletion of soil calcium and magnesium. However, the specific changes to rhizosphere nitrogen cycling under mulching and its relationship with calcium and magnesium remain unclear. To address this, our study investigated rhizosphere nitrogen cycling in Lei-bamboo across a short-term degradation cycle. We found that mulching enhanced the abundance of 16S-rRNA and nitrogen-cycling functional genes. Correspondingly, it also significantly enhanced soil respiration and nitrogen-cycling functional potentials, leading to ammonium accumulation but nitrate depletion. The rhizosphere nitrogen-cycling network reorganized as the relative contributions of *gdhA*, AOB-*amoA*, *napA*, *nirK*, and *nosZ* increased. Surprisingly, nitrite accumulated under mulching due to a decrease in the *nxrA*/AOB ratio. Although total organic carbon, total nitrogen, and pH were the main drivers of nitrogen cycling variation, magnesium also exerted considerable influence. Subsequent field amendment experiments confirmed that magnesium was the most limited nutrient under mulching. Magnesium supplementation further elevated rhizosphere microbial biomass and nitrogen-cycling functional potential, which calcium did not. The composition indicators of nitrogen-cycling groups, such as *nxrA*/AOB, as well as the content of ammonia and nitrite, reverted toward their original states after magnesium application, but the nitrate further reduced. Our findings suggest that magnesium application can partially counteract the negative effects of mulching on the rhizosphere nitrogen cycle. We discovered that nitrification processes and *nxrA* were more sensitive to magnesium than other nitrogen-cycling processes and functional genes, indicating they could be key sites where magnesium regulates nitrogen cycling. This research provides a new idea for the biogeochemical coupling of magnesium and nitrogen and offers useful guidance for sustainable mulching in Lei-bamboo agriculture.

## Graphical Abstract

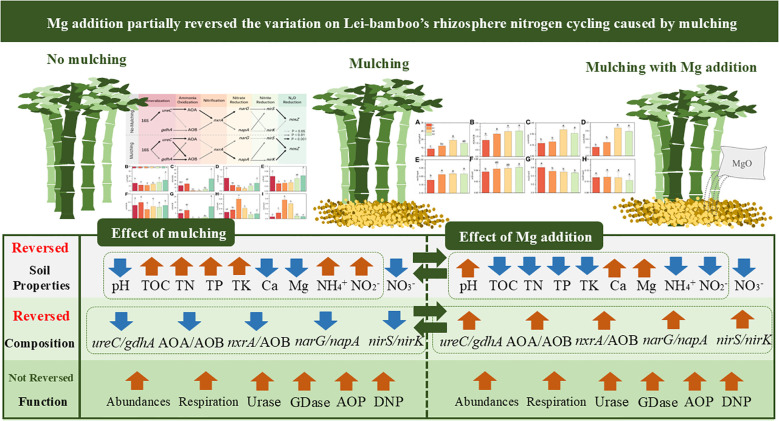

## Highlights

- Mulching altered the functional group composition and the metabolites of nitrogen cycling
- Mulching enhanced the abundance and activity of nitrogen cycling microbes
- Mg addition reversed the change on soil properties and composition but nitrate
- Mg addition further enhanced the abundance and activity of nitrogen cycling microbes
- *nxrA* and AOP were the potential key gene and process regulated by Mg

## 1 Introduction

*Phyllostachys praecox* (Lei-bamboo) is a widely planted bamboo species for shoot production in Asia, with an annual industry output exceeding 2 billion CNY (Wei et al., 1994; Wang et al., 2017; Gui et al., 2018). It serves as a pillar industry and plays a significant role in promoting regional poverty alleviation (Wei et al., 1994; Wang et al., 2017; Gui et al., 2018). In most regions, Lei-bamboo cultivation engaged intensive management that mulching in winter. The mulch materials like rice husks and straw fragments are applied to form a 30–50 cm thick layer on the soil surface, to enhance soil temperature and promote shoot emergence and yields. However, after three years of mulching, shoot production declines sharply, requiring 1–2 years of fallow for recovery. This rapid degradation has become a major constraint on the economic benefits and sustainable development of Lei-bamboo cultivation, yet the underlying mechanisms remain poorly understood (Wei et al., 1994; Zhou et al., 2017).

Nutrient imbalance induced by mulching is considered a key driving force in short-term degradation of Lei-bamboo forest (Guo et al., 2013; Chen et al., 2015). Previous research found that mulching increases phosphorus and potassium content in bamboo tissues but reduces nitrogen, magnesium, and calcium content (Zhou et al., 2024). This is primarily attributed to organic acids produced from mulching substrate fermentation, which lower soil pH and accelerate the leaching of Ca and Mg, leading them relatively limited in the rhizosphere and bamboo tissues (Boy and Wilcke, 2008; Lim et al., 2008; Clarholm et al., 2015; Li et al., 2023). Notably, Ca and Mg deficiencies, particularly Mg, may strongly correlate with reduced N utilization and yield decline (Zhou et al., 2024). The internal mechanisms that Mg influences crop nitrogen uptake capacity by regulating genes related to nitrogen absorption, transport, and storage, were widely reported (Tian et al., 2021). However, our research reveals that Ca and Mg deficiency under mulching triggers a shift in the core function of the rhizosphere microbial network from N acquisition to Ca and Mg acquisition (Zhou et al., 2024). This suggests that Ca and Mg deficiency may also alter rhizosphere N-cycling functions (Deng et al., 2012; Zhou et al., 2022), thereby affecting Lei-bamboo’s N utilization through altering soil N-metabolites composition, which may be an important external mechanism waiting to be further resolved.

While some studies confirmed that Mg significantly influences soil N-cycling processes (e.g., denitrification, N leaching, and ammonia volatilization), the results were often contradictory (Poss and Saragoni, 1992; Vestgarden et al., 2001; Wang et al., 2022; He et al., 2023; Wang et al., 2024). For instance, Mg applications have been reported to either promote (Wang et al., 2022) or suppress (He et al., 2023) nitrous oxide emissions, increase (Poss and Saragoni, 1992) or decrease (Wang et al., 2024) N leaching, and elevate or reduce soil ammonium levels (Vestgarden et al., 2001). Such discrepancies may stem from different ecosystem or vegetation type (Yousaf et al., 2021; Potarzycki et al., 2022). Lei-bamboo is predominantly cultivated in subtropical regions with acidic soils, where the silica-aluminate parent materials are recalcitrant to weathering for the release of new Ca and Mg (Wang et al., 2017; Prietzel et al., 2021). Meanwhile, the further acidification caused by over fertilization and mulching exacerbates the leach of exchangeable Ca and Mg in Lei-bamboo forests (Wei et al., 1994; Zhou et al., 2017). Hence, Lei-bamboo forest may suffer a more severe Ca- and Mg-deficient than other reported forestry ecosystems. In such contexts, Ca and Mg might play more important roles in shaping rhizosphere N-cycling functionality than macronutrients do, yet this remains understudied (Boy and Wilcke, 2008; Clarholm et al., 2015; Su et al., 2019; Yang et al., 2023). Moreover, non-N elements can participate in N-cycling modulation as stoichiometry components, redox substrates or core catalytic components of N-cycling enzymes, thus influence the abundance, composition, and activity of specific N-cycling functional groups (Krapp, 2015; Huang et al., 2017; Kappaun et al., 2018; Zhou et al., 2020; Shafiee et al., 2021; Wang et al., 2021; Bourceau et al., 2023; Shi et al., 2023; Du et al., 2024; Liu et al., 2024; Zhang et al., 2024). However, Ca and Mg, traces element that are neither redox-sensitive nor a core catalytic element in N-cycling enzymes. Thus, how Ca and Mg induces N-cycling changes warrants further investigation.

Hence, there are two key questions addressed in this study: Does Ca and Mg deficiency caused by mulching affect rhizosphere N-cycling functionality in Lei-bamboo forests? To what extent and through what mechanisms (or sites) do Ca and Mg influence N-cycling processes? To address these questions, we first investigated the changes in the abundance, composition, and activity of N-cycling functional groups during a short-term mulching-degradation cycle and identified their relationship to Ca and Mg. Then, Ca and Mg addition experiments were conducted to verify their effects on these functional groups. Given prior findings that the rhizosphere microbial network shifted toward Mg and Ca acquisition after three years of mulching, along with marked Ca and Mg deficiencies in soil and bamboo tissues, we hypothesize that (i) Ca and Mg will exert the strongest control over N-cycling functional group dynamics. (ii) Ca and Mg supplementation should reverse the shifts on nitrogen cycling caused by mulching.

## 2 Methods

### 2.1 Sites for investigation

The study site locates in Dongxiang District, Fuzhou City, Jiangxi Province. Detailed climatic conditions and agricultural managements of the sites, the sampling methods and investigation design had been depicted in our previous research. Soil samples were collected right after mulch removal in 2023, which covering a full mulching cycle: 0–3 years of mulching (M0–M3) and 1–2 years of recovery (R1–R2) (Zhou et al., 2024). During sampling, four 10m×10m fields were set in each stage, and 10 random subsamples were collected from each field, then the subsamples were thoroughly mixed and 500 g representative sample was chose for that field.

### 2.2 Amendment experiment

In the field of investigation, the sampling sites that have been mulched for 2-years were selected for amendment experiment. In March 2024, right after the mulch was removed, 16 sampling sites of 10m×10m were set, 4 of them for control (C), 4 for carbonate amendment (A1), 4 for magnesium oxide amendment (A2), and 4 for both carbonate and magnesium oxide amendment (A3). CaCO_3_ and MgO were applied in early March, mid-June, mid-September, and mid-November, which followed the conventional fertilization schedule according to the main growth stages of aboveground parts, rhizome, shoot bud, and bamboo shoots for *Phyllostachys violascens*, respectively. The 10 kg of calcium (25 kg CaCO_3_) and 10 kg of magnesium (16.7 kg MgO) were evenly applied in each treatment site to enhance the availability of Ca and Mg in soils with minimal addition of other elements and less alteration to soil pH. CaCO_3_ and MgO were applied in the form of fine powders to lead them been applicated to the field as evenly as possible and increase their availability. They were applied about 2 days before conventional fertilization to minimize the adsorptive effect of the powders to conventional fertilizers. Soil samples were collected in March 2025, immediately after mulch materials were removed, following the same protocol as previous studies.

### 2.3 Measure of soil physicochemical properties

Air-dried soil (0.5–1 g, sieved to 0.15 mm) was digested in a Kjeldahl flask with 8 mL concentrated H_2_SO_4_ and 10 drops of HClO_4_. After heating for 20–40 min until the solution turned clear, the digestate was transferred to a 100 mL volumetric flask, diluted, and filtered after overnight settling. The total phosphorus (TP) was measured via the molybdenum-antimony colorimetric method. Digestate (5–10 mL) was diluted, adjusted to pH ~3 with NaOH/H_2_SO_4_, mixed with 5 mL molybdate reagent, and absorbance read at 880 nm (Shimadzu UV1900, Tokyo). The total potassium (TK) was analyzed by atomic absorption spectroscopy (Spectrum-3803AA, Shanghai) at 766.5 nm using the digestate (Kurokawa et al., 2020).

The total organic carbon (TOC) and total nitrogen (TN) were determined using an Elementar analyzer (Berlin, Germany) (Chen et al., 2016). Samples (0.1 g) were combusted at 950 °C with a carrier gas flow rate of 250 mL/min. Soil pH was measured in a 1:2.5 (w/v) soil:water slurry using a pH meter (Leici PHS3G, Shanghai). The exchangeable magnesium (Mg) and exchangeable calcium 197 (Ca) were measured by an atomic spectrum spectrophotometer (Spectrum-3803AA, Shanghai, 198 China) The burning gas was ethyne (purity > 99.99%). A 2.0 g soil sample was placed into a 50 ml centrifuge tube and extraction solution (1 mol L^-1^ NH_4_Ac, pH=7.0) was added. Next the solution was shaken at 300 rpm for 30 min, and the supernatant collected. Content of Mg and Ca were determined at 285.2 nm and 422.7 nm, respectively (Kurokawa et al., 2020).

The ammonia, nitrite and nitrate were extracted by 2 mol L^-1^ KCl (pH=8.4, buffered by KH_2_PO_4_ and Na_2_HPO_4_), 1:4 (m:v) of soil to solution ratio, 300 rpm shaking for 10 min. The ammonia, nitrite and nitrate were measured by indophenol blue method, sulfanilamide-NEDD colorimetry and dual-wavelength UV spectrophotometry, respectively (Song et al., 2024).

### 2.4 Measure of functional potential

Respiration potential: 5g fresh soil was located in a 500 ml bottle, 40 ml 10 mmol^-1^ glucose was added, incubated under 25 °C, 300rpm for 1h, then 50 ml gas in the bottle was gathered and the CO_2_ was detected by gas-chromatograph (Agilent 7890A, America), then the respiration potential was calculated and adjusted by the water content of the soil.

Ammonia oxidation potential (AOP): 5g fresh soil was mixed with 20 ml 1 mmol^-1^ (NH_4_)_2_SO_4_ buffered solution (NaCl 8.0 g L^-1^, KCl 0.2 g L^-1^, Na_2_HPO_4_ 0.2 g L^-1^, pH 7.0), and 10 mmol^-1^ KClO_3_ was added to inhibit nitrite oxidation. Incubated under dark, 25 °C, 300rpm for 24h, the 20 ml 2 mol L^-1^ KCl was added to extract the nitrite produced, then the ammonia oxidation potential was calculated and adjusted by the water content of the soil.

Denitrification potential (DNP): 5g fresh soil was added in 500 ml bottle, 40 ml substrates were added (0.1 mmol L^-1^ KNO_3_, 1 mmol L^-1^ glucose), then sealed the bottle, changed the gas in the bottle with 90% N_2_ and 10% C_2_H_2_, incubated under 25 °C, 300rpm for 0.5h and 1.5h, and gathered 20 ml gas and stored in labco bottle (12 ml), respectively. The denitrification potential was calculated as the release rate of N_2_O.

Urase activity: 1g fresh soil was evenly distributed in the outer circle of diffusion dish, 3 ml phosphate buffer (pH 7.0), and 2 drops toluene were added, 5 ml boric acid indicator (20g H_3_BO_3_ dissolved in 1L water, 2 ml 0.1% ethanol dissolved methyl red, 10 ml 0.1% ethanol dissolved bromocresol green), adjust pH to nearly 4.8 by drop diluted H_2_SO_4_ and NaOH, apply an alkaline gel solution around the outer edge of the cover slip and gently slide the cover slip to form a narrow gap (for solution addition), introduce 5 mL of 100 g L^-1^ urea solution through the gap, then reseal the chamber tightly, fasten the cover slip firmly using a rubber band to ensure no leakage, incubate at 25 °C for 15h to allow the reaction to proceed. After incubation, perform standard acid titration on the inner chamber solution to quantify the reaction products.

Glutamate dehydrogenase activity (GDase): 5g fresh soil and 12.5 ml tris(hydroxymethyl)aminomethane buffer (pH 7.6), mixed under 25 °C, 300rpm for 1h, then centrifuged under 15000rpm for 10 min, the enzyme locates in supernatant. 1 ml supernatant was added in a 2 ml centrifuge tube, then 0.1 ml NH_4_Cl (0.1 mol L^-1^), 0.1 ml α-Ketoglutaric acid (0.06 mol L^-1^) and 0.1 ml NADPH (15 mmol L^-1^) were added, evenly mixed and incubated under 25 °C for 3h, detect the absorbance of 340 nm in 0h and 3h, respectively. The activity was measured as the reduction of NADPH.

### 2.5 The quantitative PCR

According to the protocol offered by producer, soil DNA was extracted by FastDNA-SPIN Kit for Soil (MP Biomedicals, Santa Ana, CA), and the content and quality was checked by NanoDrop Technologies Inc (Wilmington, America), stored under -80 °C. Quantitative PCR adopt 10 μL system, the upper and follower primer 0.2 μL, SYBR-Green(Takara)5 μL, Rox 0.2 μL, DNA 5 ng, dd H_2_O 10 μL, amplified by ABI-7900HT (Applied biosystem, America). The detailed primer and program information were listed in **Table 1**.

**Table 1:** Primers and programs for nitrogen functional gene qPRC.

Gene	Primer	Sequence 5’~3’	Length (bp)	Program	Reference
16S	16SrRNA-F	GGGTTGCGCTCGTTGC	107	97°C 3min,27×(95°C 30s, 55°C 30s, 72°C 45s), 4°C	(Zhu et al., 2018)
	16SrRNA-R	ATGGYTGTCGTCAGCTCGTG			
AOA	amoA1F	GGGGTTTCTACTGGTGGT	490	95°C 5min,39×(95°C 15s, 55°C 15s, 72°C 20s), 4°C	(Nguyen et al., 2018)
	amoA2R	CCCCTCKGSAAAGCCTTCTTC			
AOB	amoAF	STAATGGTCTGGCTTAGACG	660	97°C 3min,39×(95°C 10s, 57°C 10s, 72°C 15s), 4°C	(Nguyen et al., 2018)
	amoAR	GCGGCCATCCATCTGTATGT			
*nirK*	nirK-876	ATYGGCGGVCAYGGCGA	165	95°C 3min,39×(95°C 15s, 55°C 15s, 72°C 20s), 4°C	(Liang et al., 2021)
	nirK-1040	GCCTCGATCAGRTTRTGGTT			
*nirS*	cd3aF	GTSAACGTSAAGGARACSGG	400	95°C 3min,39×(95°C 10s, 57°C 10s, 72°C 15s), 4°C	(Liang et al., 2021)
	R3cdR	GASTTCGGRTGSGTCTTGA			
*narG*	narG-f	TCGCCSATYCCGGCSATGTC	173	97°C 3min,40×(95°C 60s, 58°C 60s, 72°C 60s), 4°C	(Smith Cindy et al., 2007)
	narG-r	GAGTTGTACCAGTCRGCSGAYTCSG			
*napA*	napAF	AAYATGGCVGARATGCACCC	518	70°C 3min,40×(94°C 5min, 59°C 45, 72°C 45s), 4°C	(Smith Cindy et al., 2007)
	napAR	GRTTRAARCCCATSGTCCA			
*nosZ*	nosZ-1126F	GGGCTBGGGCCRTTGCA	256	97°C 3min,40×(95°C 60s, 58°C 60s, 72°C 60s), 4°C	(Kandeler et al., 2006)
	nosZ-1381R	GAAGCGRTCCTTSGARAACTTG			
*ureC*	ureC-F	AAGSTSCACGAGGACTGGGG	317	97°C 3min,40×(95°C 60s, 58°C 60s, 72°C 60s), 4°C	(Fisher et al., 2017)
	ureC-R	AGGTGGTGGCASACCATSAGCAT			
*gdhA*	gdhA-F	GCCATCGGYCCWTACAAGGG	277	95°C 2min,34×(95°C 30s, 54°C 30s, 72°C 60s), 4°C	(Xiao et al., 2021)
	gdhA-R	GCGGTCGAGCAAATAGTCAA			
*nxrA*	F1norA	CAGACCGACGTGTGCGAAAG	322	94°C 3min,30×(94°C 30s, 55°C 45s, 72°C 45s), 4°C	(Poly et al., 2008)
	R1norA	TCYACAAGGAACGGAAGGTC			

### 2.6 Statistics

The correlation analysis and between group differences analyses (one-way ANOVA, Tukey’s HSD) were performed using IBM SPSS Statistics 27. The nitrogen cycling network of functional groups were visualized by Cytoscape v3.3.0 according to spearman correlationship metrics.

## 3 Results

### 3.1 The variation on soil physicochemical properties

The mulching treatment significantly altered the content of soil nutrients and key metabolites for nitrogen cycle (**Table 2**). Mulching gradually decreased the soil pH from 5.45 ± 0.03 (M0) to 4.25 ± 0.11 (M3). After mulch removal, the pH rebounded to 4.64 ± 0.16 (R2), which was comparable to M1 (4.77 ± 0.03). TOC peaked at 34.79 ± 2.65 g kg^-1^, more than twice of the initial level (M0: 15.47 ± 1.76 g kg^-1^), but declined to 17.76 ± 0.48 g kg^-1^ by R2. TN followed a similar trend, with 3-years mulching nearly doubling its content, from 3.07 ± 0.15 g kg^-1^ (M0) to 5.68 ± 0.14 g kg^-1^ (M3), before decreasing to 4.41 ± 0.16 g kg^-1^ (R2). TP and TK also increased under mulching, rising from 1.34 ± 0.13 g kg^-1^ (M0) and 2.48 ± 0.13 g kg^-1^ (M0) to 1.88 ± 0.12 g kg^-1^ (M3) and 3.05 ± 0.14 g kg^-1^ (M3), respectively. Unlike TOC and TN, TP remained a similar level to M3 even after mulch removal (R1: 1.69 ± 0.09 g kg^-1^; R2: 1.74 ± 0.10 g kg^-1^), while TK further increased to 3.33 ± 0.09 g kg^-1^ (R2). In contrast, exchangeable calcium and magnesium decreased significantly under mulching, dropping from 70.45 ± 4.71 mg kg^-1^ (M0) and 47.51 ± 2.80 mg kg^-1^ (M0) to 35.68 ± 2.56 mg kg^-1^ (M3) and 27.48 ± 4.53 mg kg^-1^ (M3), respectively. During recovery stage, they partially recovered to 46.63 ± 2.77 mg kg^-1^ (R2) and 37.77 ± 3.20 mg kg^-1^ (R2). Ammonia nearly doubled in the first year of mulching, increasing from 17.93 ± 1.85 mg kg^-1^ (M0) to 32.65 ± 0.84 mg kg^-1^ (M1), and further rising to 38.28 ± 1.08 mg kg^-1^ (M3). After mulch removal, it sharply declined to 21.32 ± 1.85 mg kg^-1^ (R1) and 18.55 ± 0.55 mg kg^-1^ (R2), returning to M0 levels. Nitrate was significantly reduced by mulching, decreasing from 15.80 ± 1.13 mg kg^-1^ (M0) to 8.53 ± 0.82 mg kg^-1^ (M1), with the lowest level observed at 6.92 ± 0.26 mg kg^-1^ (M3), then recovered to 11.58 ± 0.58 mg kg^-1^ (R2). Nitrite reached its highest concentration in M3 (64.53 ± 6.35 µg kg^-1^), followed by M2 (34.86 ± 3.83 µg kg^-1^), both significantly exceeding the other stages, which ranged from 13.56 ± 0.98 µg kg^-1^ (M0) to 18.44 ± 1.26 µg kg^-1^ (R1).

**Table 2:** The variation on environmental factor variation during a mulching cycle.

Stages	Ph	TOC g kg^-1^	TN g kg^-1^	TP g kg^-1^	TK g kg^-1^	Exchangeable ca mg kg^-1^	Exchangeable mg mg kg^-1^	NH_4_^+^-N mg kg^-1^	NO_3_^–^N mg kg^-1^	NO_2_^–^N ug kg^-1^
M0	5.45 ± 0.03a	15.47 ± 1.76d	3.066 ± 0.15e	1.34 ± 0.13c	2.48 ± 0.13d	70.45 ± 4.71a	47.51 ± 2.80a	17.93 ± 1.85d	15.80 ± 1.13a	13.56 ± 0.98c
M1	4.77 ± 0.03b	26.57 ± 1.62b	3.54 ± 0.13d	1.48 ± 0.14bc	2.74 ± 0.10cd	54.68 ± 1.56b	41.30 ± 3.67ab	32.65 ± 0.84b	8.53 ± 0.82cd	17.54 ± 0.69c
M2	4.31 ± 0.19c	31.44 ± 3.38a	4.69 ± 0.22b	1.69 ± 0.11ab	2.89 ± 0.11bc	55.16 ± 1.44b	33.51 ± 2.34cd	36.31 ± 1.64a	7.27 ± 0.23de	34.86 ± 3.83b
M3	4.25 ± 0.11c	34.79 ± 2.65a	5.68 ± 0.14a	1.88 ± 0.12a	3.05 ± 0.14b	35.68 ± 2.56d	27.48 ± 4.53d	38.28 ± 1.08a	6.92 ± 0.26e	64.53 ± 6.35a
R1	4.33 ± 0.11c	21.58 ± 1.50c	4.62 ± 0.11bc	1.69 ± 0.09ab	3.11 ± 0.13ab	37.78 ± 2.86d	29.31 ± 2.35d	21.32 ± 1.85c	9.05 ± 0.61c	18.44 ± 1.26c
R2	4.64 ± 0.16b	17.76 ± 0.48cd	4.41 ± 0.16c	1.74 ± 0.10a	3.33 ± 0.09a	46.63 ± 2.77c	37.77 ± 3.20bc	18.55 ± 0.55cd	11.58 ± 0.58b	15.28 ± 0.94c

The different letters indicate significant differences among groups (Tukey’s HSD P<0.05).

### 3.2 The variation on respiration and nitrogen function potentials

**Figure 1:**
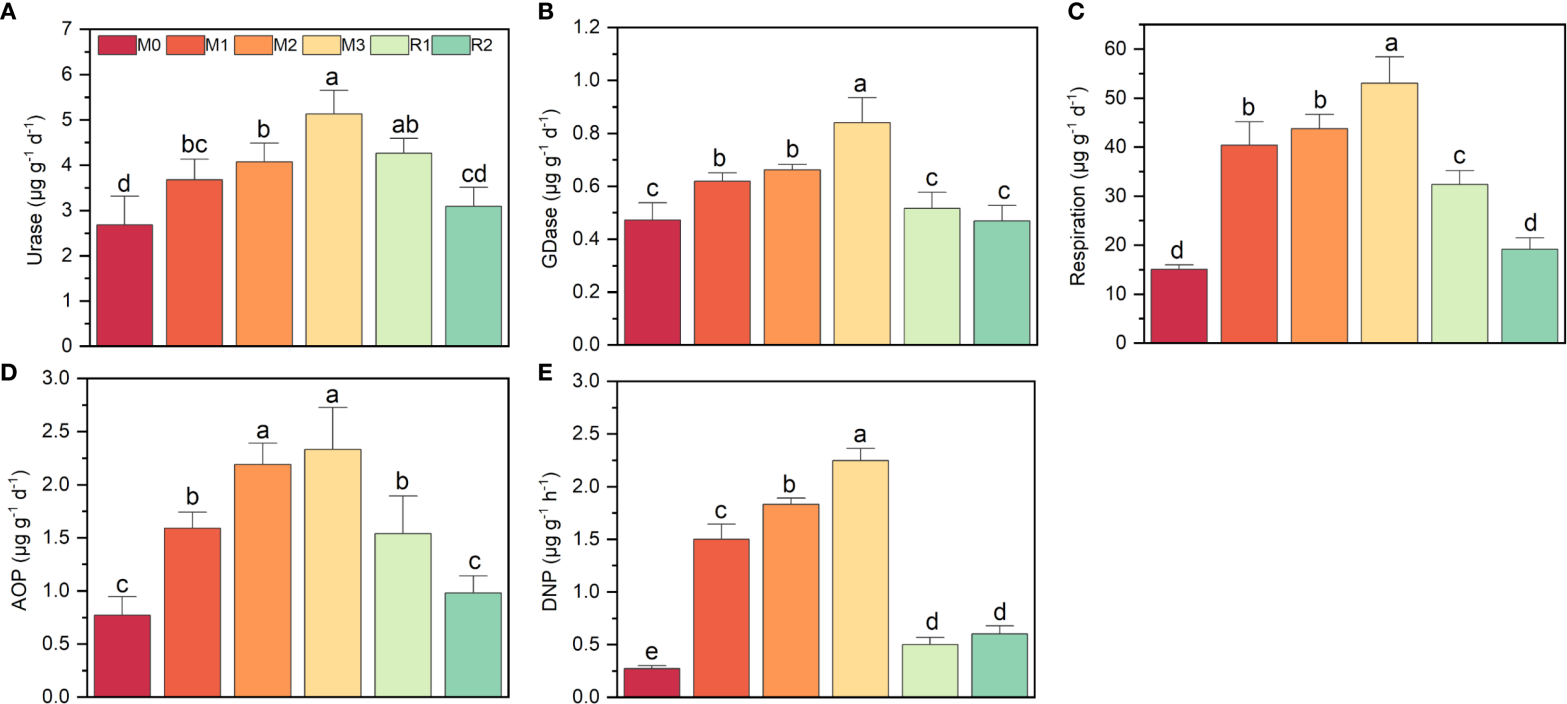
The variation on respiration and nitrogen cycling functional potentials during a mulching cycle. The subfigure **(A-E)** depicts urase activity, glutamate dehydrogenase activity, respiration, ammonia oxidation potential and denitrification potential, respectively. The different letters indicate significant differences among groups (Tukey’s HSD P<0.05). The M0, M1, M2, M3, R1 and R2 indicate waiting to be mulched, been mulched for 1 year, been mulched for 2 years, been mulched for 3 years, recovered for 1 year and recovered for 2 years, respectively.

The key enzyme activities increased significantly under mulching, though with different extent, all the potentials were reduced during recovery stage (**Figure 1**). Urase nearly doubled, rising from 2.68 ± 0.63 µg g^-1^ d^-1^(M0) to 5.13 ± 0.23µg g^-1^ d^-1^ (M3), then reduced to 3.09 ± 0.30 µg g^-1^ d^-1^ (R2) (**Figure 1A**). GDase activity showed only a modest rise of nearly 0.4-fold, from 0.47 ± 0.06 µg g^-1^ h^-1^ (M0) to 0.84 ± 0.09 µg g^-1^ h^-1^ (M3) (**Figure 1B**). AOP and respiration both increased over twofold, from 0.77 ± 0.17 µg g^-1^ d^-1^ (M0) and 15.04 ± 0.65 µg g^-1^ d^-1^ (M0) to 2.33 ± 0.33 µg g^-1^ d^-1^ (M3) and 53.03 ± 5.87 µg g^-1^ d^-1^ (M3), respectively. Then gradually reduced to level near M0 (**Figures 1C, D**). DNP exhibited the most dramatic increase, nearly eightfold, jumping from 0.27 ± 0.02 µg g^-1^ d^-1^ (M0) to 2.24 ± 0.10 µg g^-1^ d^-1^ (M3). But after mulch removal, DNP rapidly declined to only 0.50 ± 0.06 µg g^-1^ d^-1^ (M0) (**Figure 1E**).

### 3.3 The variation on the abundance, composition and network of functional groups

**Figure 2:**
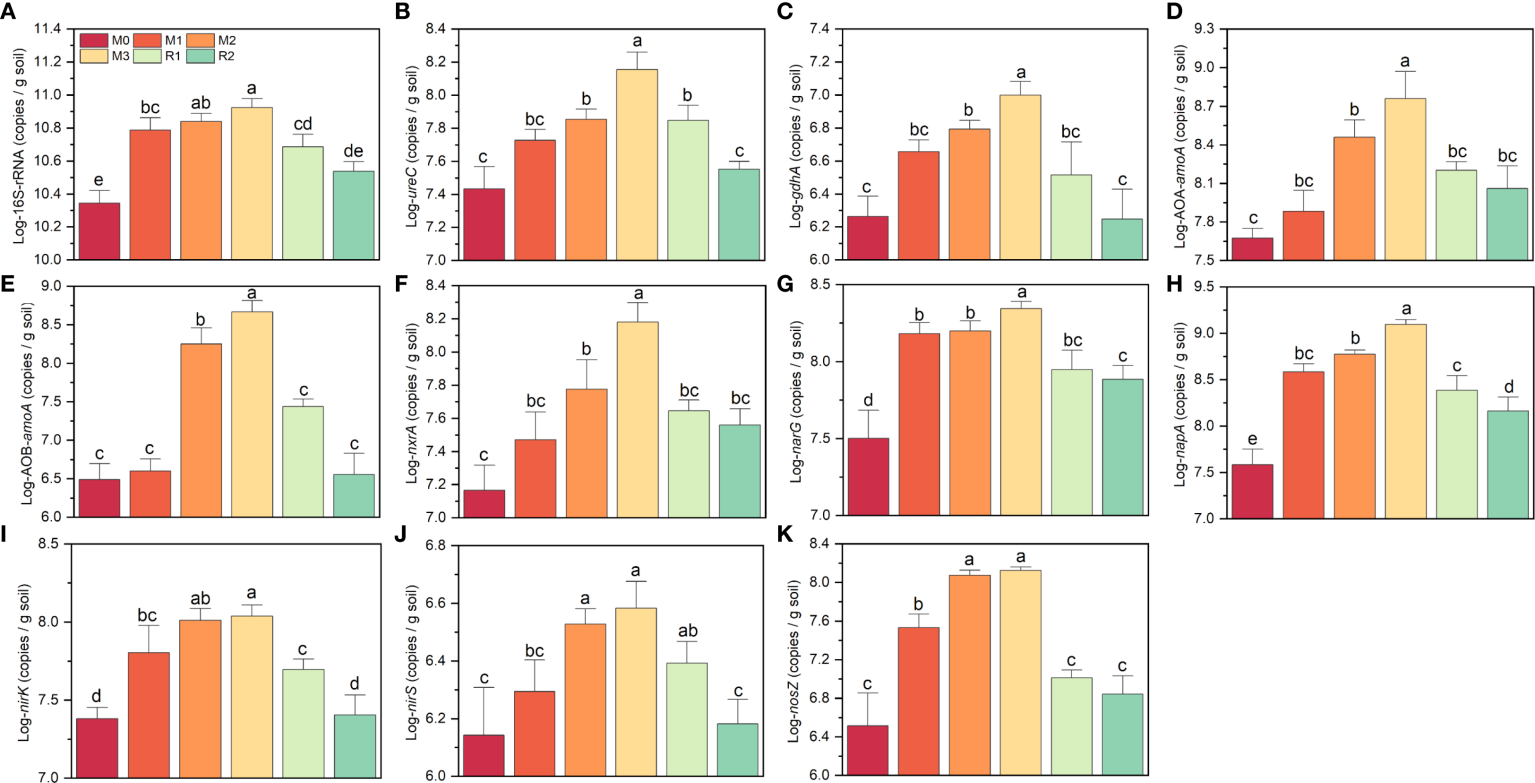
**(A–K)** indicates the variation on log transformed abundance of 16S-rRNA and nitrogen cycling related functional genes. The different letters indicate significant differences among groups (Tukey’s HSD P<0.05). The M0, M1, M2, M3, R1 and R2 indicate waiting to be mulched, been mulched for 1 year, been mulched for 2 years, been mulched for 3 years, recovered for 1 year and recovered for 2 years, respectively.

The abundance of 16S-rRNA and the nitrogen cycle-related functional genes all increased with mulching years and partially returned during recovery stage (**Figure 2**). 16S-rDNA increased by ~3.72 folds, rising from 2.27 × 10^10^ copies g^-1^ (M0) to 8.44 × 10^10^ copies g^-1^ (M3), then declining to 3.47 × 10^10^ copies g^-1^ (R2) (**Figure 2A**). *ureC* and *gdhA* were 2.79 × 10^7^ copies g^-1^ and 1.91 × 10^6^ copies g^-1^ (M0), respectively, peaked at 3.17 × 10^8^ copies g^-1^ and 2.25 × 10^7^ copies g^-1^ (M3), and then decreased to levels comparable to M0 (3.30 × 10^7^ copies g^-1^ and 8.00 × 10^6^ copies g^-1^, R2) (**Figures 2B, C**). The most dramatic increase was observed in AOB, which surged nearly 140-fold, from 3.32 × 10^6^ copies g^-1^ (M0) to 4.79 × 10^8^ copies g^-1^ (M3). In contrast, AOA only showed a 13-fold increase, rising from 4.75 × 10^7^ copies g^-1^ (M0) to 6.06 × 10^8^ copies g^-1^ (M3), *nxrA* showed a 10-fold increase, rising from 1.53 × 10^7^ copies g^-1^ (M0) to 1.56 × 10^8^ copies g^-1^ (M3) (**Figures 2D-F**). From M0 to M3, the *narG*, *napA*, *nirK*, *nirS* and *nosZ* also increased during mulching, but *napA* and *nosZ* increased respectively nearly 32 folds and 52 folds, the *narG*, *nirK* and *nirS* increased only 4.5 folds, 2.7 folds and 4.5 folds, respectively (**Figures 2G-K**). Thus, AOB, *napA* and *nosZ* were most sensitive to mulching.

**Figure 3:**
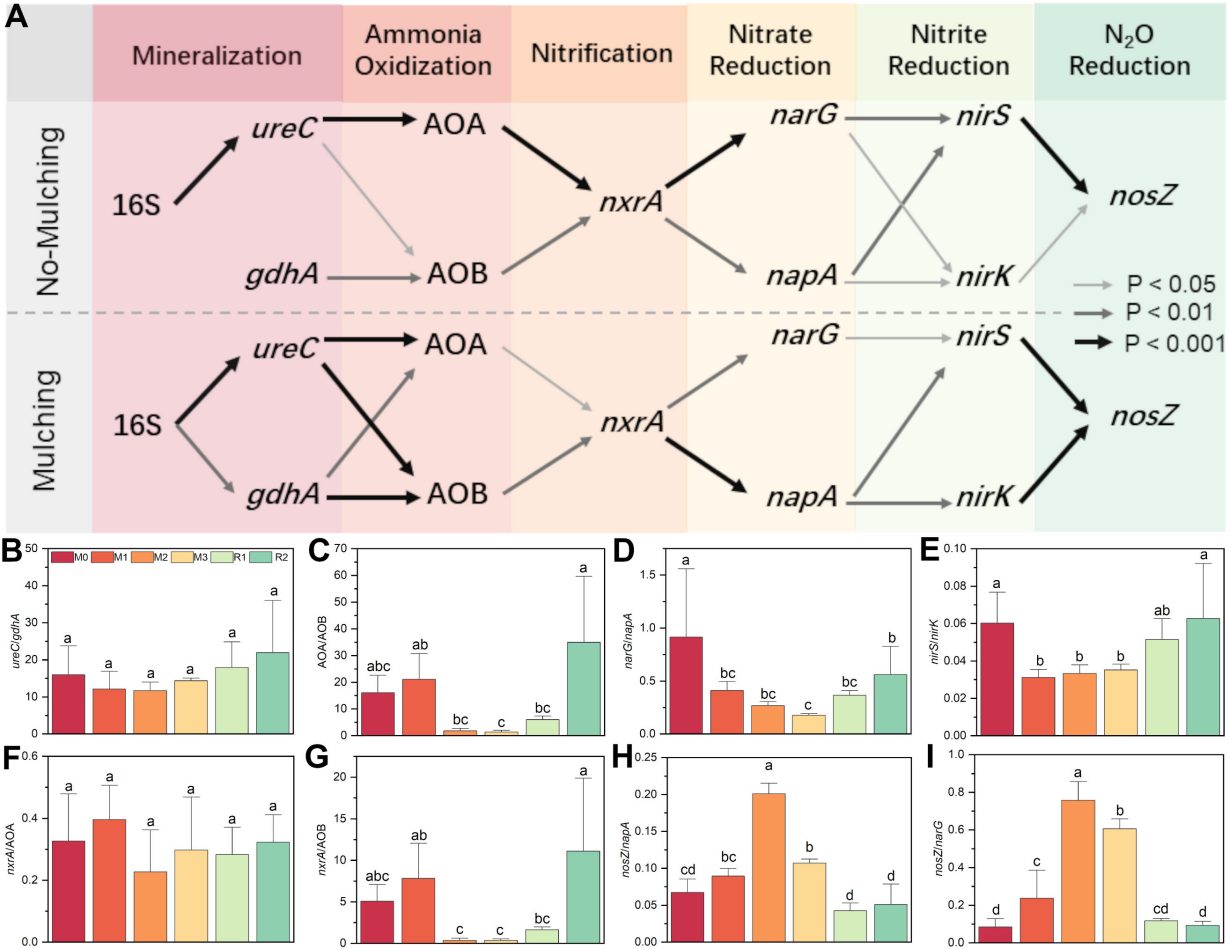
The network and composition for nitrogen cycling functional genes. The thick, common and thin arrows in subfigure **(A)** indicate significant correlated under P<0.001, P<0.01 and P<0.05 level, respectively. For subfigure **(B-I)**, the different letters indicate significant differences among groups (Tukey’s HSD P<0.05). The M0, M1, M2, M3, R1 and R2 indicate waiting to be mulched, been mulched for 1 year, been mulched for 2 years, been mulched for 3 years, recovered for 1 year and recovered for 2 years, respectively.

Mulching significantly altered the co-occurrence network of nitrogen cycle functional genes. Without mulching (M0, R1 and R2), the dominant pathway was 16S→*ureC*→AOA→*nxrA*→*narG*→*nirS*→*nosZ*. Genes such as *gdhA*, AOB, *napA*, and n*irK*, which have functional redundancy with *ureC*, AOA, *narG*, and *nirS*, also participated in the network but exhibited weaker correlations in the network. While under mulching (M1, M2 and M3), the primary pathway shifted to 16S→*ureC*/gdhA→AOB→*nxrA*→*napA*→*nirS*/*nirK*→*nosZ*. The links of 16S→*gdhA*, *gdhA*→AOB, *ureC*→AOB, *napA*→*nirK*, *nirK*→*nosZ* were strengthened by mulching. Meanwhile, the AOA→*nxrA*→*narG*→*nirS* pathway was weakened. The reorganization of the co-occurrence network indicates that mulching enhanced the relative contributions of *gdhA*, AOB, *napA*, and *nirK* in the nitrogen cycle (**Figure 3A**).

**Figure 4:**
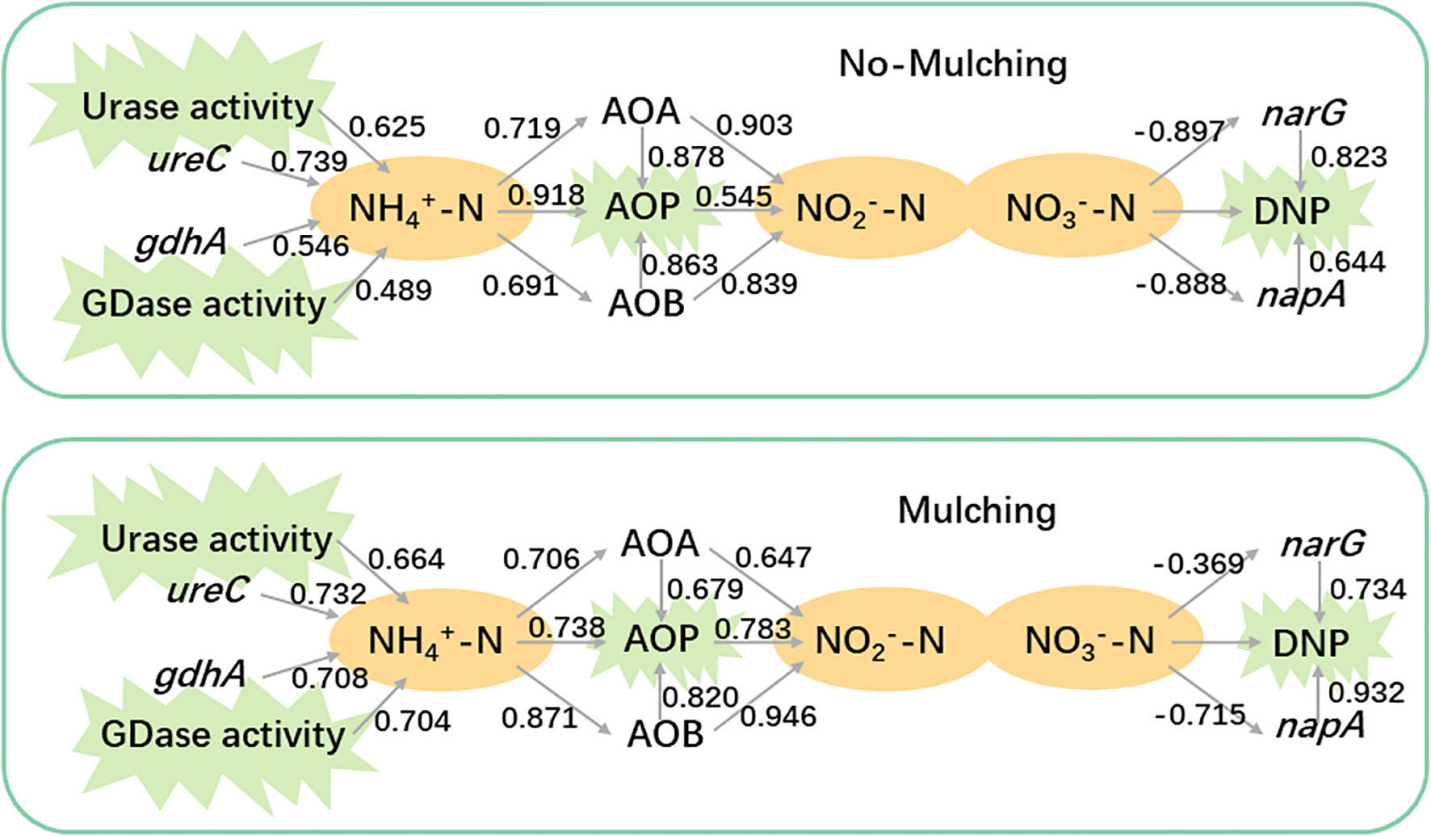
The relationship among gene abundances, nitrogen metabolite and function potentials. The number near the link indicate spearmen coefficient between the items linked.

The *ureC/gdhA* ratio was initially 15.97 ± 6.73 (M0), it slightly decreased to 11.66 ± 2.25 (M2) before gradually increasing to 21.94 ± 10.59 (R2), as mulching had a slightly stronger enhancing effect on *gdhA* than on *ureC* (**Figure 3B**). The AOA/AOB ratio started at 16.09 ± 5.87 (M0), peaked at 21.07 ± 9.78 (M1), then sharply declined to 1.80 ± 0.89 (M2) and 1.30 ± 0.49 (M3). After mulch removal, it rebounded to 5.98 ± 1.47 (R1) and 34.91 ± 17.71 (R2). This indicates that AOB abundance increased much more significantly than AOA during M2 and M3 (**Figure 3C**). The *narG/nosZ* ratio significantly decreased from 0.91 ± 0.47 (M0) to 0.41 ± 0.10 (M1), reaching its lowest point at 0.18 ± 0.01 (M3). After mulch removal, it partially recovered to 0.56 ± 0.22 (R2) (**Figure 4D**). The *nirS/nirK* ratio declined from 0.060 ± 0.019 (M0) to 0.031 ± 0.004 (M1), then slightly increased to 0.035 ± 0.003 (M3), recovered to 0.051 ± 0.014 (R1) and 0.062 ± 0.022 (R2) (**Figure 4E**). These findings also support that mulching enhances the relative dominance of *gdhA*, AOB, *napA*, and *nirK* in the nitrogen cycle.

The *nxrA*/AOA ratio did not change significantly with mulching, which fluctuating between 0.23 ± 0.11 (M2) and 0.40 ± 0.09 (M1) (**Figure 3F**). However, the *nxrA*/AOB ratio was 5.06 ± 1.88 at M0, increased slightly to 7.82 ± 3.01 (M1), then dropped sharply to 0.37 ± 0.19 (M2) and 0.35 ± 0.16 (M3). After mulch removal, it rose gradually to 1.63 ± 0.22 (R1) before increasing rapidly to 11.11 ± 6.00 (R2) (**Figure 3G**). The maximum value of *nosZ/napA* was 0.20 ± 0.02 (M2), followed by 0.11 ± 0.005 (M3), both significantly higher than in non-mulching stages (M0, R1, and R2) (**Figure 3H**). Similarly, *nosZ/narG* was 0.76 ± 0.10 (M2) and 0.61 ± 0.04 (M3), significantly higher than in non-mulching stages, while the minimum was only 0.09 ± 0.02 (R2).

Integrated analysis of functional potential, metabolites, and functional gene abundances revealed that mulching enhanced the contributions of GDase, AOB, and *napA* to the nitrogen cycle (**Figure 4**). Under non-mulching conditions, urase and the *ureC* gene exhibited higher correlation coefficients with ammonium (0.625 and 0.739, respectively) compared to GDase and *gdhA* (0.489 and 0.546). Under mulching, the correlations of urase and *ureC* with ammonium remained similar (0.664 and 0.732), whereas those of GDase and *gdhA* increased to 0.704 and 0.708, indicating an enhanced contribution of GDase mediated processes to ammonium production. The correlation between ammonium and AOA was not significantly affected by mulching. However, the correlation with AOB increased from 0.691 (non-mulching) to 0.871 (mulching). Under non-mulching conditions, AOB showed slightly lower correlations with AOP and nitrite (0.862 and 0.839, respectively) compared to AOA (0.878 and 0.903). In contrast, under mulching, AOB exhibited stronger correlations (0.863 with AOP and 0.946 with nitrite nitrogen), while AOA’s coefficient decreased to 0.679 and 0.647, suggesting that mulching enhanced the role of AOB in AOP. For denitrification genes, *narG* displayed high negative and positive correlations with DNP under non-mulching conditions (0.823), but these decreased under mulching (0.734). Conversely, *napA* showed correlations of 0.644 with DNP under non-mulching conditions, which strengthened to 0.932 under mulching, surpassing *narG*. This indicates that mulching favored the contribution of *napA* over *narG* in denitrification processes.

### 3.4 The environmental drivers for N-cycling variation

**Figure 5:**
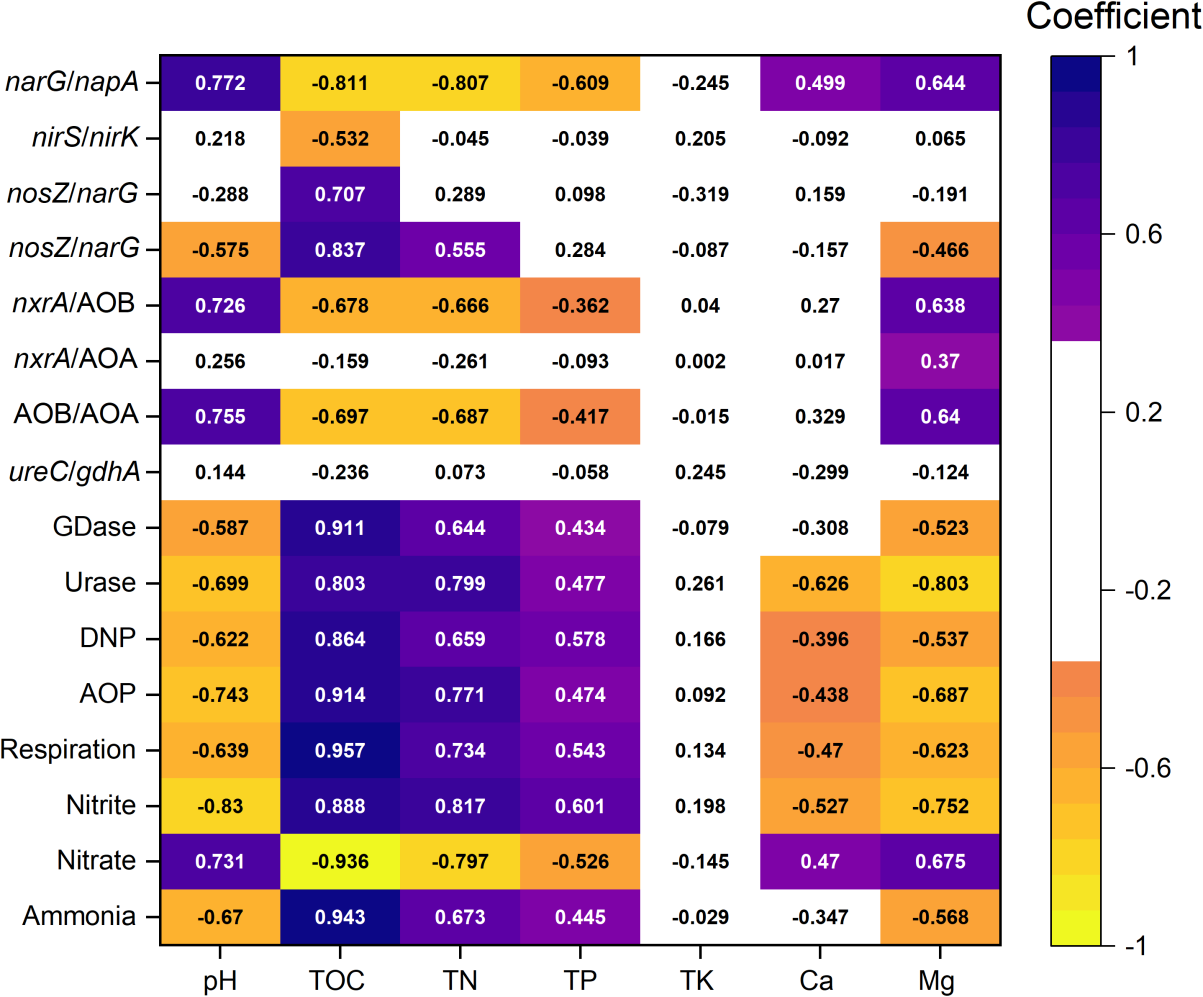
Correlation between major environmental factors and indicators of nitrogen cycle intermediate metabolites, functional potentials, and functional community composition. Colored sections represent significant Spearman correlations (P < 0.05), while white sections indicate non-significant correlations (P > 0.05).

The indicators most strongly correlated with nitrogen cycle functional potentials and intermediate metabolites remained TOC, TN, and pH. Mg also exhibited significant correlations with most functional potentials and metabolites. Though Mg was not the most influential factor, its impact was consistently greater than that of TP and TK (**Figure 5**). TOC was strongly correlated with GDase (0.911), urase (0.803), DNP (0.864), AOP (0.914), respiration (0.957), nitrite (0.888), nitrate (-0.936), ammonium (0.943). TN and pH showed a lower coefficient to them, and Mg and TP showed further weaker but still significant coefficient. For denitrification-related functional composition indicators (e.g., *narG/napA, nirS/nirK, nosZ/napA, nosZ/narG*), TOC showed the strongest correlations. pH, TN, and Mg also had significant but slightly weaker correlations with *narG/napA* and *nosZ/narG*, where Mg ranked below pH and TN but above TP. Mg was the only environmental factor significantly correlated with all nitrification-related community composition indicators (*nxrA*/AOA, *nxrA*/AOB, AOA/AOB). Though pH had the highest correlation with *nxrA*/AOA and AOA/AOB, TOC, TN, and Mg also showed a slightly weaker coefficient (ranging between 0.638–0.697). In contrast, mineralization-related community composition indicators (*ureC/gdhA*) did not show significant correlations with any environmental factors. Notably, Mg exhibited stronger correlations with nitrogen cycle functions and community composition than Ca.

### 3.5 The variation on soil physicochemical properties and nitrogen cycling with Ca and Mg application

The addition of calcium carbonate and magnesium oxide significantly altered the physicochemical properties of the degraded Lei-bamboo forest soil. These changes were primarily reflected in a notable increase in pH, a significant decrease in TN, TP, ammonium, nitrate, and nitrite. But TK was not significantly varied (**Table 3**). Among these, nitrite exhibited the most pronounced change, that calcium application reduced its content by nearly one-quarter, magnesium application by more than half, and their combined application by almost three-quarters. In most cases, calcium and magnesium demonstrated synergistic effects, though magnesium’s influence was more pronounced, particularly regarding soil nitrogen components such as ammonium, where magnesium induced more drastic changes than calcium. Yet, the sole addition of calcium carbonate slightly increased TOC, while magnesium alone had no significant effect, yet their combined application led to a reduction in TOC.

**Table 3:** The variation on soil physicochemical properties with Ca and Mg application.

Treatments	Ph	TOC g kg^-1^	TN g kg^-1^	TP g kg^-1^	TK g kg^-1^	Exchangeable ca mg kg^-1^	Exchangeable mg mg kg^-1^	NH_4_^+^-N mg kg^-1^	NO_3_^–^N mg kg^-1^	NO_2_^–^N ug kg^-1^
C	4.13 ± 0.10c	35.10 ± 1.67ab	5.61 ± 0.19a	1.83 ± 0.14a	3.09 ± 0.25a	35.88 ± 1.72b	28.39 ± 3.80a	36.43 ± 1.58a	7.30 ± 0.26a	59.76 ± 6.12a
A1	4.38 ± 0.02b	36.03 ± 1.59a	5.10 ± 0.20b	1.82 ± 0.10ab	3.24 ± 0.07a	73.81 ± 7.24a	27.16 ± 2.09a	32.18 ± 1.06b	6.66 ± 0.20a	50.98 ± 6.06b
A2	4.47 ± 0.02ab	33.90 ± 2.20ab	4.70 ± 0.14b	1.71 ± 0.04ab	3.11 ± 0.12a	37.70 ± 1.95b	64.79 ± 3.25b	28.40 ± 0.78c	5.64 ± 0.15b	29.88 ± 12.71c
A3	4.54 ± 0.04a	32.16 ± 1.62b	4.69 ± 0.26b	1.68 ± 0.06b	3.04 ± 0.10a	78.32 ± 5.40a	68.24 ± 3.45b	27.84 ± 1.31c	5.33 ± 0.13b	17.67 ± 6.87d

The different letters indicate significant differences among groups (Tukey’s HSD P<0.05).

**Figure 6:**
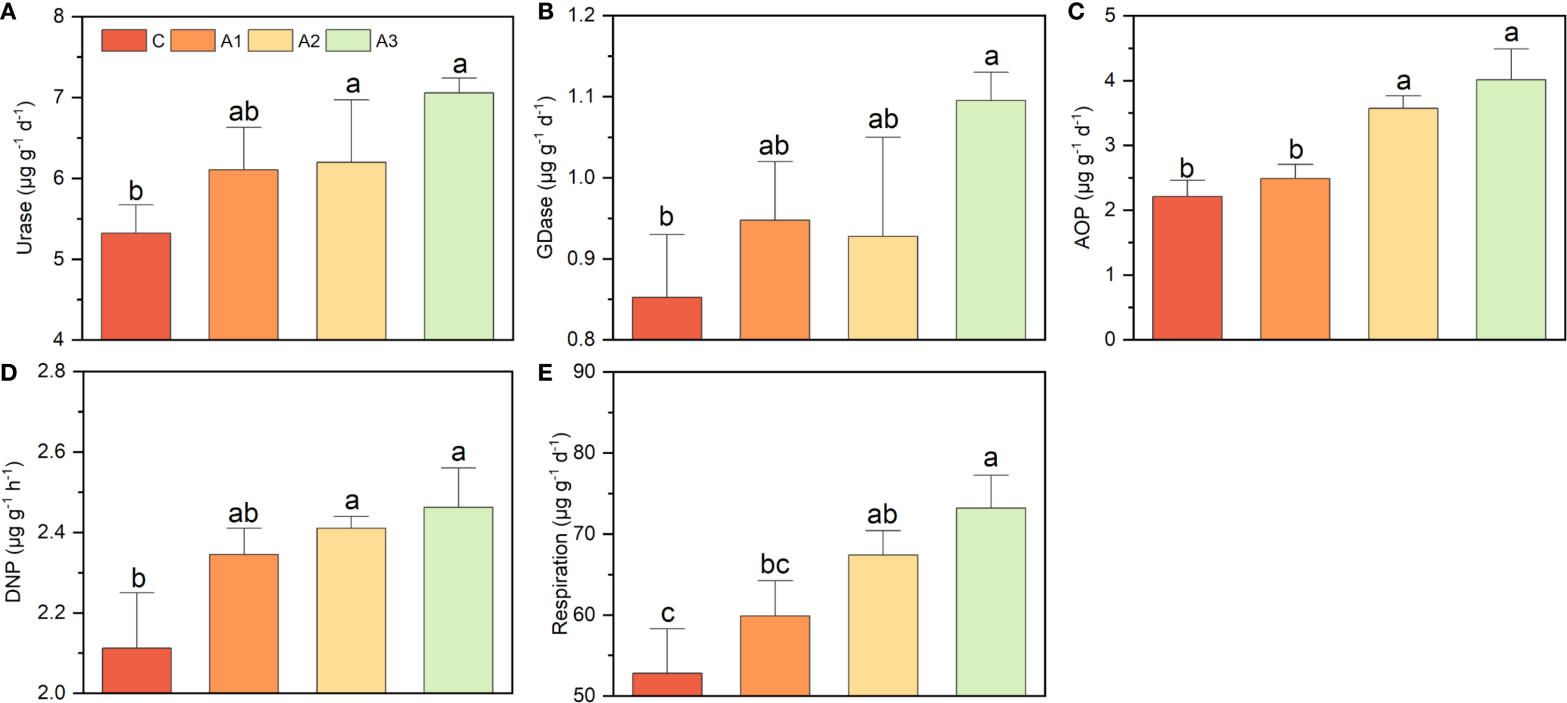
**(A–E)** indicates the effects of calcium and magnesium addition on key functional potentials. C, A1, A2, and A3 represent the control group, Ca-only, Mg-only, and Ca+Mg combined treatments, respectively. Different lowercase letters above the bars indicate significant differences among groups (Tukey’s HSD, P < 0.05).

The key functional potentials were enhanced to different extent by calcium and magnesium addition, with the combined application showing greater improvement than individual treatments (**Figure 6**). For example, urase activity and respiration potential increased by nearly 40% under the Ca+Mg treatment, whereas individual Ca or Mg application only raised them by approximately 20%. Similarly, GDase activity increased by nearly 30% under the combined treatment, while individual applications only showed an insignificant enhancement. AOP exhibited the strongest response to Mg addition, as the control and Ca-only groups measured 2.23 ± 0.21 µg g^-1^ d^-1^ and 2.49 ± 0.23 µg g^-1^ d^-1^, respectively, whereas the Mg-only and Ca+Mg treatments reached 3.57 ± 0.14 µg g^-1^ d^-1^ and 4.01 ± 0.37 µg g^-1^ d^-1^, representing an increase of over 60%. In contrast, DNP showed only a modest rise from 2.11 ± 0.10 µg g^-1^ h^-1^ to 2.46 ± 0.09 µg g^-1^ h^-1^ (only about 16.5%) under Mg addition.

**Figure 7:**
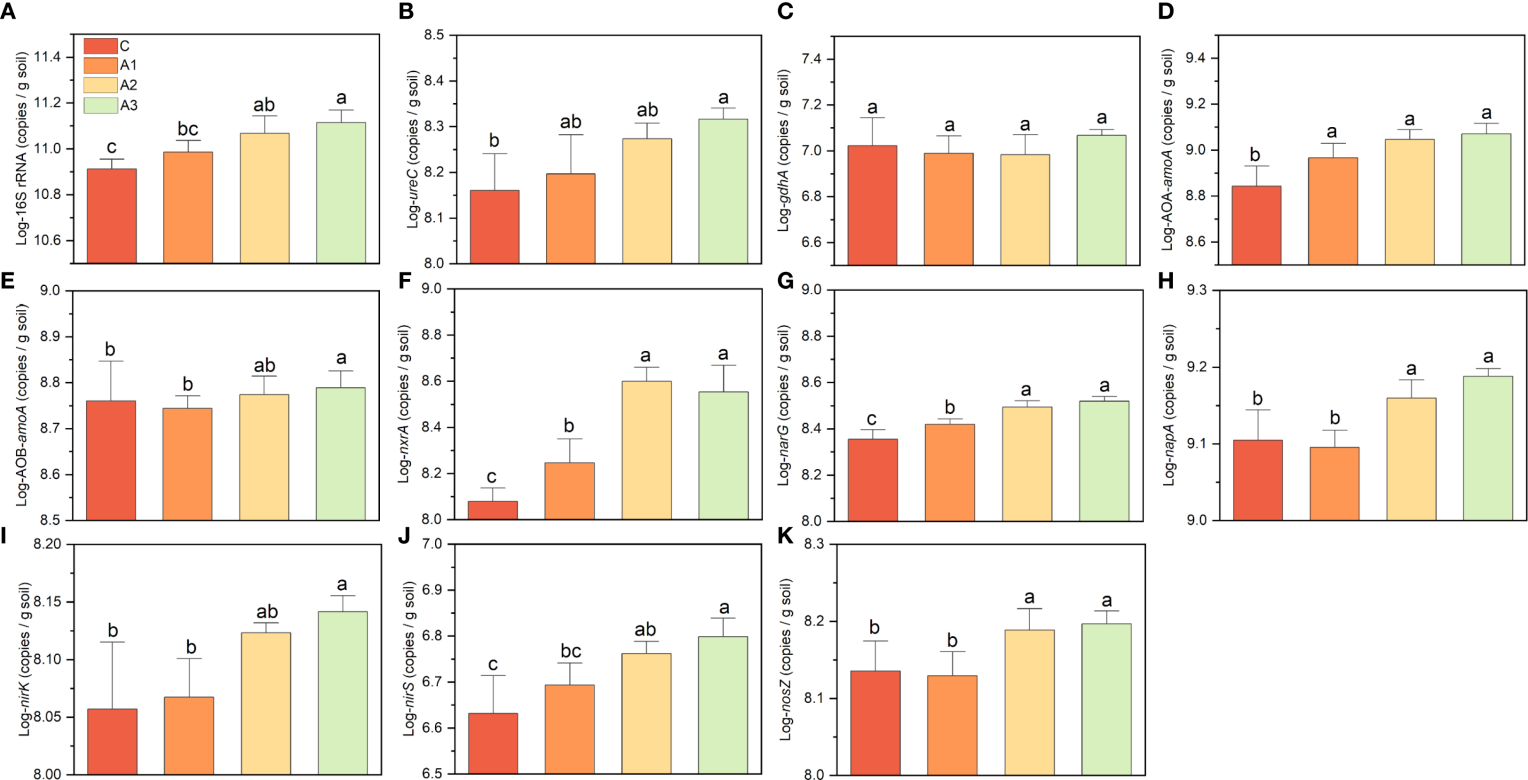
**(A–K)** indicates the variation on key functional gene abundances under calcium and magnesium addition. C, A1, A2, and A3 represent the control group, Ca-only, Mg-only, and Ca+Mg combined treatments, respectively. Different lowercase letters above the bars indicate significant differences among groups (Tukey’s HSD, P < 0.05).

The addition of calcium and magnesium increased the abundance of 16S rRNA and nitrogen-cycling functional genes (except *gdhA*), with magnesium being the primary driver (**Figure 7**). Except for *nxrA* and *narG*, calcium alone did not significantly enhance functional gene abundance. The combined application of Ca+Mg slightly increased gene abundance compared to Mg alone, but the difference was not statistically significant. When comparing A3 with C, 16S rRNA gene abundance increased by ~0.5-fold. Most nitrogen-cycling genes showed lower extent increases than 16S, with enhancement levels ranked as follows: AOB (0.06-fold), *gdhA* (0.09-fold), *nosZ* (0.15-fold), *nirK* (0.20-fold), *napA* (0.21-fold), *ureC* (0.41-fold), *nirS* (0.45-fold), *narG* (0.46-fold) and AOA (0.68-fold). The most pronounced increase was observed for *nxrA*, which rose by 2.03-fold. These findings suggest that magnesium plays a dominant role in boosting microbial functional gene abundance, while calcium only contributed slightly unless combined with Mg. The nitrite-oxidizing gene (*nxrA*) exhibited the strongest response, indicating a potential shift in nitrogen transformation processes under Ca+Mg amendment.

**Figure 8:**
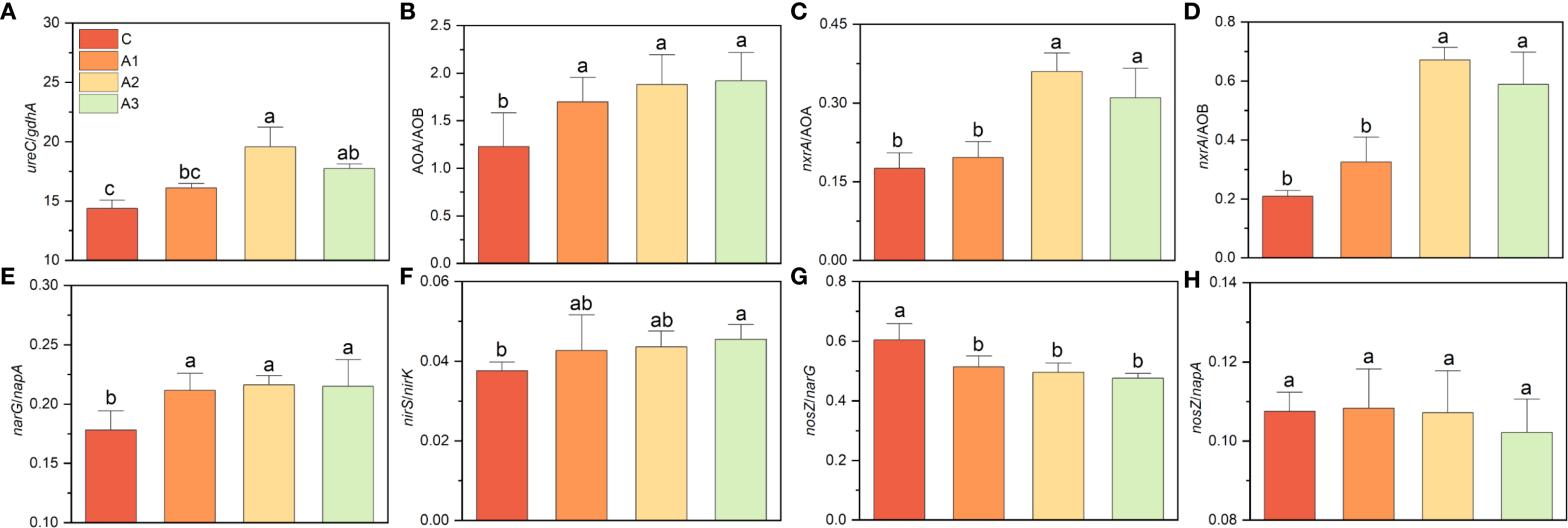
**(A–H)** indicates the variation on nitrogen cycling functional group composition under calcium and magnesium addition. C, A1, A2, and A3 represent the control group, Ca addition only, Mg addition only, and combined Ca+Mg addition, respectively. Different lowercase letters above the bars indicate significant differences among groups (Tukey’s HSD, P < 0.05).

The differential responses of nitrogen-cycling functional genes to Ca/Mg application inevitably led to changes in the composition of functional groups (**Figure 8**). The ratio of *ureC/gdhA* increased significantly by nearly one-third under Mg addition (from 13.77 in C to 19.55 in A2), and AOA/AOB also increased from 1.23 (C) to 1.96 (A3). The *nxrA*/AOB ratio rose from 0.175 (C) to 0.359 (A2), but Ca addition did not cause a significant change; a similar trend was observed for *nxrA*/AOA. Both Ca and Mg addition significantly increased *narG/napA* and *nirS/nirK* while decreasing *nosZ/narG*, though no significant differences were found among A1, A2, and A3. In contrast, *nosZ/napA* showed no significant change.

## 4 Discussion

### 4.1 Variation on nitrogen-cycling function under mulching and the driving factors

First, the 16S abundance, N-cycling gene abundance, and related activities were nearly all increased by mulching, indicates that organic matter released from mulch materials and applied fertilizers created a favorable environment for rhizosphere microbial communities, serving as the primary driver of N-cycling functional shifts (**Figures 1**, **2** and **5**). Our previous studies also found that mulching enhanced rhizosphere microbial α-diversity and increased network complexity, further supporting this view (Zhou et al., 2024). Though his contrasts with reports suggesting that mulching contributes to yield decline and soil microbial community degradation in Lei-bamboo forests (Guo et al., 2013; Zhang et al., 2019), many studies demonstrate that mulching practices (e.g., straw return, livestock manure compost cover) could effectively regulate soil microbial abundance/composition and enhance nutrient-cycling activity, serving as common strategies to improve soil quality and mitigate yield degradation (Fu et al., 2021; Shinde et al., 2022; Liu et al., 2023). Thus, we argue that the observed decline in Lei-bamboo N uptake under mulching cannot be simplistically attributed to rhizosphere microbial “degradation”, but need to focus on the functional connection between rhizosphere community and roots and check whether their cooperation was loosed by mulching (Pausch et al., 2024).

Second, TOC, TN and acidification caused by mulching were the main driving force for rhizosphere nitrogen cycling variation. AOB, *napA*, and *nirK* are widely reported to be more competitive under nutrient-rich conditions. In contrast, AOA are more tolerant of low-ammonia and low-organic-matter environments, and their autotrophic growth may even be inhibited when ammonia or organic matter levels are too high (Shen et al., 2012; Hink et al., 2017). Compared to *narG*, *napA* is not only involved in dissimilatory nitrate reduction but also assimilatory nitrate reduction (Coelho and Romão, 2015), and it thrives in high-organic-matter environments and boosts microbe growth (Ji et al., 2013; Chen et al., 2022). Similarly, *nirK* prefers environments with higher nitrogen and phosphorus content compared to *nirS* (Azziz et al., 2017; Liang et al., 2021). Therefore, the observed reduction in the abundance of these AOA/AOB, *narG/napA*, and *nirS/nirK* reflects the dominant role of organic matter released from mulch and fertilization in driving shifts in microbial community structure. Moreover, *nosZ* is sensitive to oxygen concentration, and the proportion of denitrifying functional groups increases under hypoxia (Kuypers et al., 2018). The rise in *nosZ/narG* and *nosZ/napA* ratios with increased mulching suggests enhanced respiration, leading to more anerobic conditions. This implies that rhizosphere microbes or bamboo roots may face oxygen deficiency (Mbukwa et al., 2023). Notably, the influence of Mg on the composition of nitrifying functional groups is comparable to that of TOC and TN, indicating that nitrification may be more sensitive to Mg than denitrification and mineralization processes.

Lastly, mulching increased soil ammonium but decreased nitrate (**Table 1**). The N forms are not only linked to plant N preference but also affect soil N loss potential and flux, serving as key indicators of soil N supply capacity (Du et al., 2024). Studies suggest that a balanced ammonium:nitrate ratio optimizes Lei-bamboo growth (Ye and Chen, 2017), implying that reduced nitrate under mulching may hinder N utilization. Notably, we observed unexpected nitrite accumulation (**Table 2**). Nitrite is highly reactive, particularly in acidic soils, where it rapidly reacts with organic matter or ammonium and disappears accordingly (Van Cleemput and Samater, 1995). However, nitrite exhibits high production fluxes in subtropical forest soil despite the low content (Yang et al., 2018). Improved extraction methods can better capture the nitrite dynamics in acidic soils (Song et al., 2024). Beyond autotrophic nitrification and denitrification, heterotrophic nitrification contributes significantly to nitrite production in acidic soils, with estimated production rates of 1.29–2.77 mg kg^-1^ d^-1^ and consumption rates of 5.84–6.74 mg kg^-1^ d^-1^. Despite higher lab-measured consumption rates, background soil nitrite levels could still be measurable (7 μg kg^-1^), though far lower than our measurements (13.56–64.53 μg kg^-1^) (Yang et al., 2018). Heterotrophic nitrification, though poorly quantified and mechanistically unclear, has been widely discovered in bacteria and fungi cells when oxidizing labile N-containing organics (e.g., amino acids) (Martikainen, 2022). Given that mulching elevated 16S copy numbers and respiration, heterotrophic nitrification and nitrite production may have increased (Martikainen, 2022). Nitrite consumption occurs via oxidation to nitrate, denitrification, dissimilatory nitrate reduction, anammox, or chemodenitrification (Van Cleemput and Samater, 1995; Yang et al., 2018; Dai et al., 2020). The significant decline in *nxrA*/AOB under mulching indicates a reduced nitrite oxidation capacity relative to ammonia oxidation, potentially promoting nitrite accumulation (Poly et al., 2008). Thus, mulched Lei-bamboo soils likely exhibit strong nitrite flux. Nitrite possesses strong oxidative capacity under acidic conditions, leading to the formation of reactive nitrogen species (RNS). These compounds cause direct damage to the membrane systems of both rhizosphere microorganisms and root cells of Lei-bamboo, thereby compromising rhizosphere microecological functions and impairing fine root physiology (Oke, 1966). Furthermore, nitrite nitrogen under acidic conditions generates gaseous nitrous acid (HONO), which influences atmospheric hydroxyl radical (·OH) concentrations and contributes to alterations in air quality (Song et al., 2023). Additionally, nitrite nitrogen can react directly with ammonium nitrogen (NH_4_^+^-N) to form nitrogen gas (N_2_) (Oke, 1966). Both processes reduce the residence time of nitrogen fertilizers in soil and lower nitrogen use efficiency (NUE). Thus, the accumulation of nitrite nitrogen may lead to declined productivity and degradation of Lei-bamboo through multiple mechanisms. However, further research is needed to clarify nitrite production/consumption processes in mulched systems (Van Cleemput and Samater, 1995; Yang et al., 2018; Dai et al., 2020).

Contrary to our first hypothesis, TOC, TN, and pH, rather than Mg, Ca, TP and TK, were identified as the main factors influencing nitrogen-cycling in the rhizosphere of Lei-bamboo under mulching. However, the effect of Mg remained stronger than that of macronutrients like P and K, also larger than Ca, and should not be overlooked.

### 4.2 Effects of calcium and magnesium application on nitrogen cycling in the rhizosphere of Lei -bamboo

Following Ca-Mg amendment, microbial biomass and activity continued to increase during the mulching period, indicating that mulching had induced Ca and Mg limitation (mainly Mg) in the soil microbial community. Previous studies have also found that mulching enhances Mg limitation from a stoichiometric perspective (Zhou et al., 2024). Magnesium is a crucial component of ATP-related catalytic enzymes and plays a vital role in energy metabolism (Tomita et al., 2017). Host can even regulate the abundance and activity of symbiotic microbes by controlling Mg supply (Cunrath and Bumann, 2019; Blanc-Potard and Groisman, 2021). Although Ca also increased the abundance of some functional genes, its effect was weaker than that of Mg. Studies in karst regions have identified Ca as a key limiting factor for soil nitrogen-cycling functions (Yang et al., 2023). However, in red soil regions, Mg is more prone to leaching and is less abundant in parent material, resulting in stronger Mg limitation compared to Ca (Boy and Wilcke, 2008; Clarholm et al., 2015; Su et al., 2019).

The application of magnesium partially counteracted the effects of mulching on the composition of nitrogen-cycling functional groups, ammonium and nitrite, consistent with previous correlation analyses (**Figures 6**–**8**; **Table 3**). This further confirms that Mg limitation induced by mulching is a prominent factor influencing nitrogen cycling (Zhou et al., 2024). While some studies have reported that Mg application affects processes such as nitrous oxide emissions, ammonia volatilization, and microbial nitrogen assimilation, the underlying mechanisms remain unclear (Poss and Saragoni, 1992; Vestgarden et al., 2001; Wang et al., 2022; He et al., 2023; Wang et al., 2024). Beyond its role as a limiting factor, it remains to be investigated whether Mg directly influences nitrogen cycling by serving as a catalytic center for specific enzymes inside of cell or indirectly through interactions with carbon and phosphorus cycling (Krapp, 2015; Huang et al., 2017; Kappaun et al., 2018; Zhou et al., 2020; Shafiee et al., 2021; Wang et al., 2021; Bourceau et al., 2023; Shi et al., 2023; Du et al., 2024; Liu et al., 2024; Zhang et al., 2024). We observed a reduction in nitrite levels following Mg application (**Table 3**). Nitrite generates reactive oxidative substances and leads oxidative stress in microbe cells (Cunrath and Bumann, 2019; Kuang et al., 2022). Magnesium has been widely reported to play a prominent role in reducing intracellular ROS of plant and animal cells, this maybe a potential reason (Wolf et al., 2008; Cai et al., 2019; Đurić et al., 2023). However, nitrate also declined instead of increasing, which was same to mulching effects. On the one hand, this is likely due to enhanced nitrogen assimilation by both microorganisms and Lei-bamboo after alleviating Mg limitation. The reduction in both free nitrate and ammonia pools supported that Mg and Ca application improved nutrient uptake efficiency by Lei-bamboo, which was further supported by the significant decrease in soil TN and TP (**Table 3**). On the other hand, although Mg and Ca application increased AOP in a higher proportion than DNP, the absolute increment in DNP was still much higher than AOP (**Figures 6C, D**), which indicate the consumption, rather than production in nitrate were faster stimulated by Mg and Ca application.

Compared to other functional processes, AOP showed more pronounced enhancement under magnesium application, this indicates that the nitrite oxidation step in the nitrification process may be a key site where magnesium affects nitrogen cycling in the Lei-bamboo rhizosphere (**Figure 7**). The *nxrA* exhibited a greater increase than AOA and AOB after magnesium supplementation (**Figure 8**). The *nxrA* is the gene responsible for encoding the subunit of nitrite oxidoreductase, which is present in nitrite-oxidizing bacteria, anoxygenic photosynthetic bacteria, and anaerobic ammonium-oxidizing bacteria (anammox) (Poly et al., 2008). Its catalytic core contains iron, and there are few reports on the mechanism about magnesium promotes *nxrA*. Our study confirms that its sensitivity to magnesium may be higher than that of other nitrogen-cycling functional microorganisms, which was supported by several research. A bacterial pure culture addition experiment demonstrated that magnesium significantly improves the heavy metal resistance of microbial nitrogen-cycling functions, increases the efficiency of microbial nitrate and nitrite removal capability, and promotes bacterial growth (He et al., 2019). A research on domestic wastewater found that magnesium has a stronger regulatory effect on the activity of nitrifying functional groups than on denitrifying groups (Zhang et al., 2020). When the magnesium ion concentration ranges from 1.1 to 3.0 mmol L^-1^, it significantly enhances nitrification (Zhang et al., 2020). Magnesium application may influence the abundance and activity of *nxrA* through multiple pathways. First, as a limiting nutrient, magnesium directly promotes biomass production (Yang et al., 2021), organic matter decomposition (Giachetti and Vivanco, 2024), and microbial community interactions (Chen et al., 2017), thereby indirectly affecting nxrA via microecological network effects. Second, magnesium enhances the scavenging of reactive nitrogen species within microbial cells, which may improve the metabolic capacity of microorganisms to process nitrite nitrogen (Bourret et al., 2017). Finally, magnesium can stimulate the anaerobic ammonium oxidation (anammox) process under high moisture conditions (Shi et al., 2025). Given that the soil in mulched Lei-bamboo forests typically exhibits high humidity (Wei et al., 1994; Zhou et al., 2017), and considering the key catalytic role of the *nxrA* gene in anammox (Chicano et al., 2021), this may represent another potential mechanism by which magnesium regulates *nxrA* in the rhizosphere of Lei-bamboo. However, more studies on cellular-to-community level are still needed to elucidate the direct relationship between magnesium and *nxrA* (He et al., 2019; Zhang et al., 2020).

Hence, partially consistent with the second hypothesis, calcium-magnesium application only partially reversed the mulch-induced changes in nitrogen cycling characteristics, mainly in terms of functional group composition and nitrogen components, but not abundances and activities.

### 4.3 Future research

The following aspects still require further investigation: On one hand, isotope tracing combined with laboratory incubation experiments should be employed to explore the dynamic characteristics of nitrogen components in the Lei-bamboo rhizosphere, particularly to elucidate the specific pathways of nitrite nitrogen accumulation and the corresponding microbial ecological mechanisms (Yang et al., 2018; Martikainen, 2022). On the other hand, it is necessary to distinguish the contribution from magnesium’s indirect effects (via altering rhizosphere nitrogen cycling) and direct effects (via altering plant physiological state), in order to comprehensively understand how magnesium promotes nitrogen uptake in Lei-bamboo through both exogenous soil processes and endogenous plant processes (Tian et al., 2021).

## 5 Conclusions

Mulching not only significantly increased the abundance of 16S rRNA and nitrogen cycling functional genes in the rhizosphere of Lei-bamboo, but also enhanced their functional potential. It also elevated the relative contributions of *gdhA*, AOB, *napA*, and *nirK* in the N cycle and altered the N cycling network. TOC, TN and pH were the primary drivers of these changes, though magnesium also exerted a considerable influence. Mulching accumulated ammonium and nitrite while reduced nitrate in the soil. The accumulation of nitrite was associated with a decreased *nxrA*/AOB under mulching, and Mg had a greater impact on this process than phosphorus and potassium. The application of Ca and Mg, particularly Mg, increased the abundance of 16S rRNA genes, N-cycling functional genes, and their functional potential, indicating that rhizosphere microbes were limited by Mg. Meanwhile, Ca and Mg application reduced ammonium, nitrite and nitrate and enhancing the contributions of *ureC*, AOA, *narG*, and *nirS*. This study confirms that Mg limitation induced by mulching significantly influences N cycling in the rhizosphere of Lei-bamboo forests, with nitrification processes and the *nxrA* gene likely serving as key regulatory points in the N cycle’s response to Mg, and Mg application can partially reverse the negative changes in soil N components and N-cycling functional community composition. Unlike previous research focusing on how Mg alters plant N uptake capacity from physiological sight, this study focuses on how Mg modulate soil N nitrogen dynamic and supply potential, which provides new knowledge about biogeographical Mg-N coupling and offers a scientific basis for optimizing N utilization by Mg application in Lei-bamboo forest.

## Data Availability

The original contributions presented in the study are included in the article/**Supplementary Material**. Further inquiries can be directed to the corresponding author.
