# Supplementary material for: Magnesium application partially reversed the negative effects of mulching on rhizosphere nitrogen cycling in a Phyllostachys praecox forest
Source: Front Plant Sci. 2025 Oct 8;16:1670128. doi: 10.3389/fpls.2025.1670128 (PMC12542910; doi:10.3389/fpls.2025.1670128)
Supplement: Supplementary file 2 [file Table2.docx]

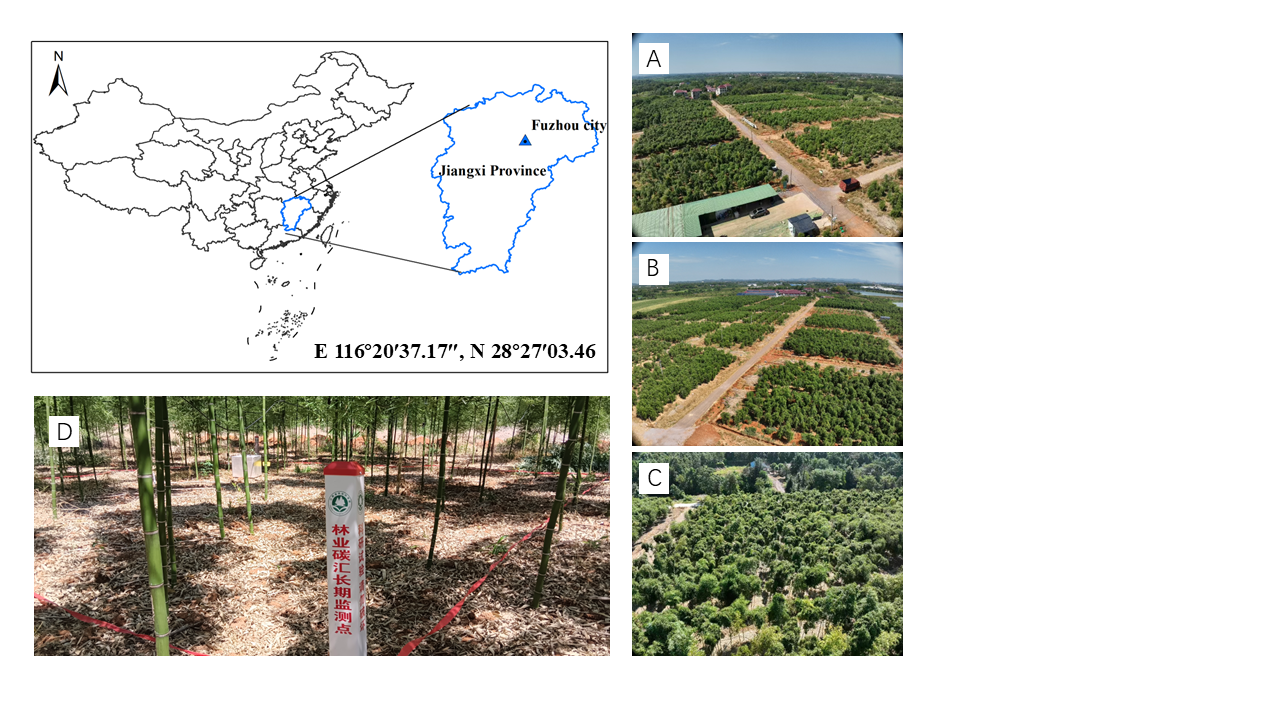


Figure S1 The sampling sites, A, B and C depicts the sites of M0~R2, M0 (A Left), M1 (B Left), M2 (A Right), M3 (B Right), R1 (C Left) and R2 (C Right). D depicts an amendment experiment sampling site.
